# Observed contrast changes in snow cover phenology in northern middle and high latitudes from 2001–2014

**DOI:** 10.1038/srep16820

**Published:** 2015-11-19

**Authors:** Xiaona Chen, Shunlin Liang, Yunfeng Cao, Tao He, Dongdong Wang

**Affiliations:** 1 State Key Laboratory of Remote Sensing Science, School of Geography, Beijing Normal University, Beijing 100875, China; 2 Department of Geographical Sciences, University of Maryland, College Park 20742, USA

## Abstract

Quantifying and attributing the phenological changes in snow cover are essential for meteorological, hydrological, ecological, and societal implications. However, snow cover phenology changes have not been well documented. Evidence from multiple satellite and reanalysis data from 2001 to 2014 points out that the snow end date (D_e_) advanced by 5.11 (±2.20) days in northern high latitudes (52–75°N) and was delayed by 3.28 (±2.59) days in northern mid-latitudes (32–52°N) at the 90% confidence level. Dominated by changes in D_e_, snow duration days (D_d_) was shorter in duration by 5.57 (±2.55) days in high latitudes and longer by 9.74 (±2.58) days in mid-latitudes. Changes in D_e_ during the spring season were consistent with the spatiotemporal pattern of land surface albedo change. Decreased land surface temperature combined with increased precipitation in mid-latitudes and significantly increased land surface temperature in high latitudes, impacted by recent Pacific surface cooling, Arctic amplification and strengthening westerlies, result in contrasting changes in the Northern Hemisphere snow cover phenology. Changes in the snow cover phenology led to contrasting anomalies of snow radiative forcing, which is dominated by D_e_ and accounts for 51% of the total shortwave flux anomalies at the top of the atmosphere.

Snow cover over the Northern Hemisphere (NH) plays a crucial role in the Earth’s hydrology and surface energy balance, and modulates feedbacks that control variations of global climate^1^. Previous studies found that snow cover in the NH has decreased significantly, i.e., the June snow cover extent (SCE) has decreased nearly twice as rapidly as the widely acknowledged September loss of sea ice extent from 1979 to 2011^2^. This trend will continue in the future according to climate projections^3^,^4^. It coincides with north hemispheric warming and is indicative of a positive feedback of surface reflectivity on the climate^3^,^5^,^6^,^7^, which may affect the global biological and ecological systems further^8^,^9^.

In a warmer climate, the date of snow melt will advance in time^10^. Earlier snow melting can lead to major alterations in timing and volume of spring snowmelt runoff, with a possible increase in the incidence of catastrophic events such as spring flooding and summer droughts. Moreover, snow cover is tightly correlated with certain biological and ecological phenomena that depends on accumulated temperature, such as plant growth and animal migrations. During winter, snow cover is beneficial to agriculture by conserving the heat of the surface, and thus, protecting the crops (mainly winter wheat) from the cold air. In high latitudes, warmer winter events damage the vegetation and reduce the following season crop yield^11^. Earlier snowmelt may reduce the availability of grass in its most nutritious form if animal migrations are not timed accordingly. In addition, changes in snow cover phenology may influence the seasonality in the terrestrial system through changes in the soil thaw and freeze dates^12^; as well as, in a warmer climate, result in permafrost degradation^13^.

Previous efforts have proved that snow cover phenology has remarkably changed in response to climate change. Reported effects at a local and regional scale include a shortening of snow duration days (D_d_)^14^,^15^, an earlier snowmelt onset^16^ and an earlier snow end date (D_e_)^14^,^17^. However, further work is still needed since published estimates of snow cover phenology mainly focused on the Northern pan-Arctic regions and conclusions were made from in-situ observations, model simulations or using a single source of images. Further studies are currently needed at a continental scale in order to better understand the changes in snow cover phenology, the mechanisms driving them, and their impacts on the Earth’s climate system.

Recent studies have revealed significant changes in the land surface temperature induced by the vanishing cryosphere^18^,^19^,^20^,^21^, as well as the increased winter precipitation induced by changes in atmospheric circulation^22^ and human activity^23^,^24^. In particular, the near-surface at the NH high latitudes is warming at rates double of those at lower latitudes, due to the combined rapid loss of sea ice and snow cover in spring and summer^20^. In spite of the climate warming on average, an ostensibly large number of high-impact cold extremes have occurred in the mid-latitudes of NH over the past decade^25^. Snow cover phenology is highly sensitive to changes in temperature and precipitation. Therefore, understanding how these climate change events featuring the high spatial heterogeneity influence the snow cover phenology has meaningful consequences for water management, sustainable development of ecosystems, and prediction of catastrophic climate related events. Thus, the objective of this study is to quantify and understand the spatial and temporal changes of snow cover phenology and to point out their causes and consequences. This knowledge is critical for assessing and projecting the future climate.

Five snow datasets are analyzed (Supplementary Table S1) in this study, including a reanalyzed dataset of daily snow depth generated by the Canada Meteorological Center (CMC)^26^, a binary daily snow cover mask derived from the Interactive Multi-sensor Snow and Ice Mapping System (IMS)^27^ and the Northern Hemisphere Weekly Snow Cover and Sea Ice Extent (NHSCE)^27^,^28^, 8-Day Level 3 snow cover fraction products (MOD10C2) derived from the Moderate Resolution Imaging Spectroradiometer Satellite (MODIS)^29^ and the snow water equivalent (SWE) derived from the Near-real-time Ice and Snow Extent (NISE) dataset^30^. A multi-data approach^5^ is employed in order to develop a combined snow cover phenology matrix that integrates snow cover phenology information detected from multiple sources of snow observations (see Methods, Supplementary Tables S2 and S3). Based on the seasonal cycle of snow cover over the NH^31^, we defined the snow cover accumulation season from the previous year (t−1) November to the current year (t) February, and the snow cover melting season from March to June of current year (t). Regarding daily snow observations, the snow onset date (D_o_) is defined as the first five consecutive days on which snow was observed to cover the ground surface in accumulation season; D_e_ is defined as the last five consecutive days when snow cover was observed in the melting season. D_d_ is defined as the number of days from the onset date of snow cover to the end date of snow cover. The specific definitions of D_o_, D_e_, and D_d_ for each dataset are described in Methods.

## Results

### Observed changes in the snow cover phenology

**Figure 1:**
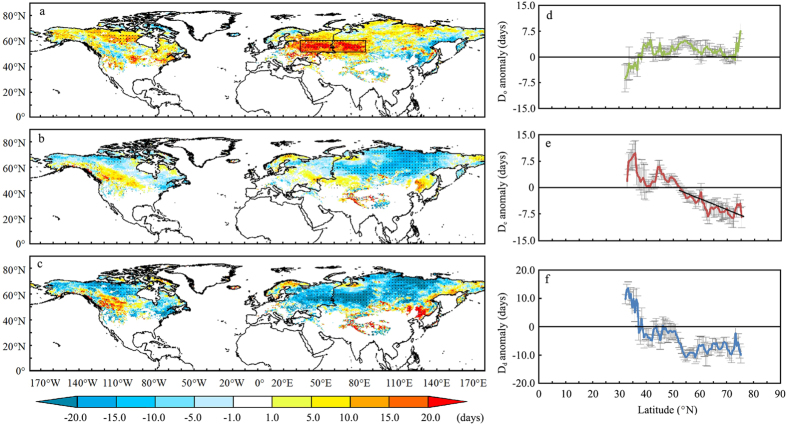
Changes of snow cover phenology over the NH from 2001 to 2014 derived from multi-data sets. Changes of snow onset date D_o_ (**a,d**), snow end date D_e_ (**b,e**) and snow duration days D_d_ (**c,f**) over the NH snow covered landmass, over 14 years. Changes are derived from the linear slope multiplied by the time span. Black dots in (**a–c**) indicate that the changes are significant at 90% CL. The zonal distribution in (**d–f**) are mapped at a 0.5 degree resolution in latitude. The error bars in (**d–f**) are calculated using equation (1) in Methods. The figure was created using *ArcGIS*^42^.

Based on the NHSCE dataset, the NH presents no noticeable changes in D_o_, an earlier D_e_ at 95% confidence level (CL), as well as an overall shorter D_d_ between the winters of 1972/73 and 2007/08^14^. However, monthly SCE anomalies from 1972 to 2014 over the NH indicate that there is a significant reduction of SCE in summer and a notable increase of SCE in winter, especially after the year 2000 (Supplementary Fig. S1). Moreover, our results pointed out a contrast in the snow cover phenologies of middle and high latitudes over the NH in recent years. The snow onset date D_o_ (Fig. 1a,d) over most parts of the NH high latitudes (40–70°N) was delayed by approximately 2.19 (±1.63) days except in latitudes lower than 40°N where D_o_ advanced by 2.70 (±1.97) days. The most notable delay of D_o_ (17.36 ± 6.67 days) occurred over Eastern Europe and Western Asia (the boxed area, Fig. 1a). Unlike the D_o_, the snow end date D_e_ (Fig. 1b,e) has advanced by about 9.66 (±2.35) days over high latitudes in Eurasia, Canada and the high elevation Tibet Plateau (TP) regions, but it was delayed by about 10.67 (±2.35) days in the mid-latitudes of Eastern Asia and Central North America (Fig. 1b). Combined spatial and temporal changes in D_o_ and D_e_ led to an extension of snow cover duration days D_d_ (Fig. 1c,f) by over 10 days in latitudes below 40°N. At regions above 40°N, D_d_ shortened at a rate of 0.7 days decade^−1^, from 2001 to 2014.

D_e_ and D_d_ are strongly correlated (≥99% CL, Pearson correlation analysis) (*r* = 0.89), and the correlation between D_o_ and D_d_ (*r* = 0.64) was also significant at a 95% CL. Attending to the small changes observed in D_o_ (Supplementary Fig. S7), changes in D_d_ in the recent decades were mainly attributed to changes in
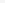
D_e_.

In addition, yearly-averaged D_e_ decreased significantly (95% CL) over the NH snow covered landmass from 2001 to 2014 (Supplementary Fig. S7). Moreover, there were large differences in zonally averaged D_e_ anomalies between regions located in middle and high latitudes (Fig. 1b,e). The sign of the zonally averaged changes in D_e_ turns at around 52°N. The average change in D_e_ below 52°N was of 3.28 (±2.59) days, with maximum delays distributed around 35.5°N (9.78 ± 3.49 days). On the other hand, changes in D_e_ averaged at −5.11 (±2.20) days in the 52–75°N range, with maximum negative changes occurring at 72 °N (−8.59 ± 2.78 days). From 35.5°N poleward, D_e_ advances linearly with latitude.

**Figure 2:**
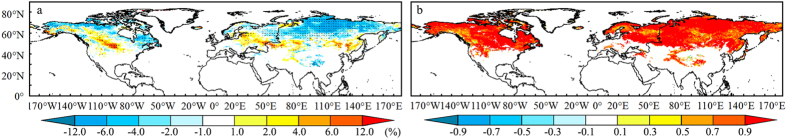
Changes in land surface albedo during the melting season. (**a**) Changes in surface albedo (%) during the melting season over 14 years and (**b**) correlation with D_e_ over the NH from 2001 to 2014. Black dots in (**a**) indicate that changes are significant at a 90% CL. The figure was created using *ArcGIS*^42^.

Furthermore, the contrasting changes in D_e_ (Fig. 1b) observed between the NH middle and high latitudes are highly similar to the spatiotemporal distribution of changes in land surface albedo (a_s_) observed during the melting season over the corresponding period ranging from 2001 to 2014 (Fig. 2a). The coefficients of linear correlation between the melting season D_e_ and a_s_ are above 0.8 over 91% of the studied area (Fig. 2b). In response to the earlier D_e_ observed in high latitudes during the melting season, a_s_ decreased due to lower land surface reflectivity, which resulted in the land-atmosphere system absorbing additional energy. On the other hand, delayed D_e_ observed in mid-latitudes during the melting season, resulted in opposite changes in a_s_, which enhanced the cooling effects of the snow cover.

## Discussion

### Attribution of snow cover phenology changes

**Figure 3:**
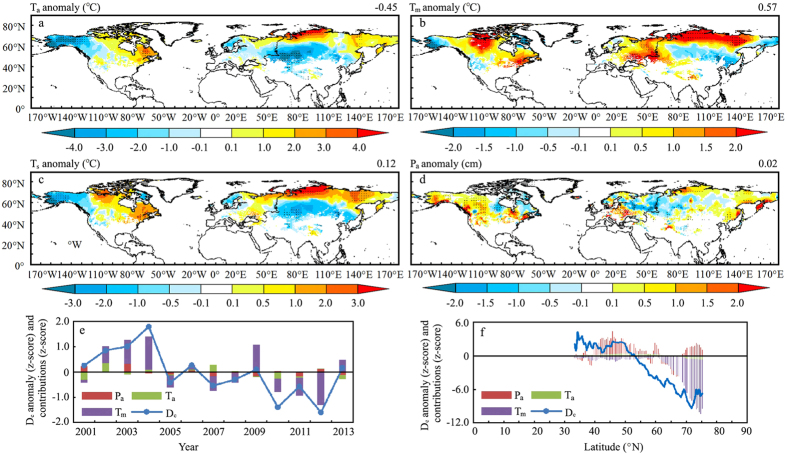
Attributions of snow cover phenology changes over the NH from 2001 to 2013. Changes in (**a**) temperature during the accumulation temperature, T_a_, (**b**) temperature during the melting season, T_m_, (**c**) temperature over the entire snow season, T_s_ and (**d**) precipitation during the accumulation season, P_a_, are derived from a linear regression model over the NH snow covered landmass from 2001 to 2013. (**e**) Yearly averaged snow end date D_e_ anomalies and contributions from T_a_, T_m_ and P_a_. (**h**) Zonally averaged D_e_ anomalies and contributions from T_a_, T_m_ and P_a_. Black dots in (**a–d**) indicate that changes are significant at a 90% CL. The figure was created using *ArcGIS*^42^.

The attribution analysis was carried out for the snow seasons from 2001 to 2013 limited by the availability of the Climatic Research Unit (CRU) Time Series (TS) dataset^32^. The analysis of linear correlations of snow onset date D_o_ with land surface temperature (T_a_) and precipitation (P_a_) over the snow accumulation season (November-December-January-February) demonstrated that the D_o_ was largely determined by T_a_ anomalies, pointing to a sensitivity of 0.28 days °C^−1^, as P_a_ was not statistically correlated with D_o_ at a 95% CL (Supplementary Figs S8 and S9). The analysis of spatial patterns showed a general positive sensitivity of D_o_ to T_a_ and negative sensitivity to P_a_ (Supplementary Fig. S10). D_o_ was most significantly sensitive to T_a_ at around 40°N, as a result of the significant decrease in T_a_ in Eastern Europe and Western Asia (Fig. 3a). This spatial distribution is basically consistent with the observed long-term tendency of large-scale cooling trends of land surface temperature in winter over the mid-latitudes^20^,^33^. The decline of winter temperatures in mid-latitudes is attributed to changes in the Arctic system through changes in storm tracks, the Jet Stream, planetary waves and their associated energy propagation^20^. In addition, the recent Pacific Ocean cooling effect also contributed to a decrease of temperature in the mid-latitudes, since the tropical cooling effect on the extra-tropics is most pronounced in winter^33^.

Changes in snow end date D_e_ were highly correlated with T_a_, land surface temperature during melting season (March-April-May-June) (T_m_) and P_a_ at a 95% CL, but T_m_ dominated (Fig. 3e). The D_e_ varies with T_a_, T_m_, and P_a_ at −0.03 days °C^−1^, −0.31 days °C^−1^ and 0.10 days cm^−1^, respectively (Supplementary Fig. S11). The observed delays of D_e_ around 45°N were largely controlled by decreased T_m_ (Fig. 3b) as well as increased P_a_ (Fig. 3d) in this region, in which the magnitude of P_a_ was the main controlling factor of D_e_ (Fig. 3f). This pattern coincides with previous findings concerning large scale cold snaps, heavy snowfall and glacier events across the United States, Europe and East Asia^25^,^34^,^35^. Increased precipitation in the mid-latitude was attributed to afforestation^24^ and strengthening westerlies^22^,^35^. In addition, the advanced D_e_ around 70°N was largely explained by the poleward increase of T_m_ (Fig. 3f).

Snow duration days D_d_ anomalies were highly correlated in space and time with temperature over the entire snow season (T_s_) and P_a_ at the 95% CL (Supplementary Figs S9 and S12). Both T_a_ and T_m_ significantly increase poleward above 60°N and decrease at different rates in the mid-latitudes (Fig. 3b, Supplementary Fig. S8b), which results in the observed contrasting T_s_ anomalies in middle and high latitudes (Fig. 3c). Contrasting T_s_ over the NH was mainly driven by Arctic amplification effects, as the warming magnitude at high latitudes was about two times as that of lower latitudes^20^,^21^. The rapid Arctic warming has contributed to dramatic melting of Arctic sea ice and spring snow cover, which is coincided with a period of ostensibly more frequent extreme weather events across the Northern Hemisphere mid-latitudes, including severe winters^20^. D_d_ varies with T_s_ and P_a_ at −0.85 days °C^−1^ and 0.14 days cm^−1^, respectively. The analysis of spatial patterns pointed out the overall negative sensitivity of D_d_ to T_s_ and its positive sensitivity to P_a_ (Supplementary Fig. S12). Even though changes in D_d_ are highly correlated with changes in T_s_, correlations of D_d_ with T_m_ and T_a_ are not statistically significant.

### Radiative forcing and albedo feedbacks of snow cover phenology changes

Land surface change is expected to contribute substantially to warming trends in the NH high latitudes^36^. To quantify the snow radiative forcing (S_n_RF) and albedo feedback induced by snow cover phenology changes, both the radiative kernel method^37^,^38^,^39^ and the Clouds and Earth’s Radiant Energy System (CERES) observations at the top of atmosphere (TOA) were employed in this study. The radiative kernel approach allows to separate the radiative response to different climate parameters and to decompose the feedback into radiative and climatic response components. In this study, the albedo radiative kernel was used to evaluate the instantaneous perturbation to the Earth’s shortwave (SW) anomaly induced by snow cover phenology changes. The CERES observations were used to study the snow cover phenology anomaly induced radiative forcing in total TOA forcing anomalies.

**Figure 4:**
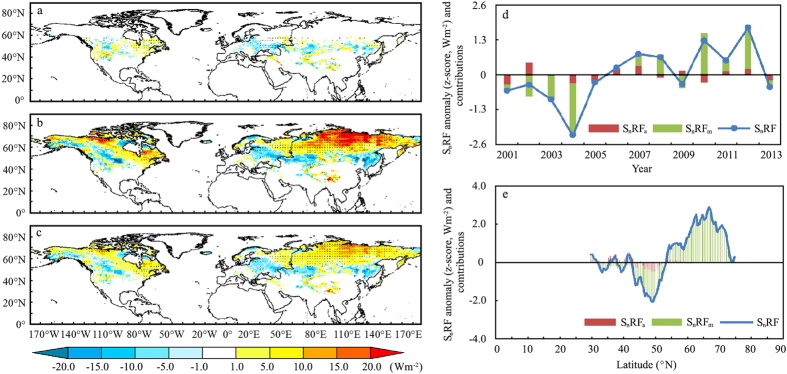
Spatiotemporal distribution of snow radiative forcing S_n_RF (in Wm^−2^) and contributions from the accumulation and the melting seasons from 2001 to 2013 using the radiative kernel approach. Estimates of S_n_RF induced by (**a**) changes in the snow onset date D_o_, (**b**) the snow end date D_e_, and (**c**) the snow duration days D_d_ from 2001 to 2013. (**d**) S_n_RF and contributions from the accumulation season S_n_RF_a_ and the melting season S_n_RF_m_. (**e**) Zonally averaged S_n_RF and contributions from the S_n_RF_a_ and S_n_RF_m_. Value in (**a–c**) were averaged from two S_n_RF_a_, S_n_RF_m_, and S_n_RF results obtained using the Community Atmosphere Model (CAM3) and the Atmosphere Model 2 (AM2), respectively. Black dots in (**a–c**) indicate that changes are significant at a 90% CL. The figure was created using *ArcGIS*^42^.

Applying the radiative kernel approach with equation (2), we inferred that changes in S_n_RF induced by the snow season variability from 2001 to 2013 were recorded to be 0.16 (±0.004) Wm^−2^ over the NH. Distinct anomalies are observed at middle and high latitudes, where changes in the snow radiative forcing during the accumulation season (S_n_RF_a_) was of 0.01 (±0.001) Wm^−2^ and changes during melting season (S_n_RF_m_) was of 0.31 (±0.011) Wm^−2^. Compared with changes in S_n_RF_a_, those in S_n_RF_m_ are the main contributor to changes in the S_n_RF in both space and time, (Fig. 4d,e). As displayed in Fig. 4c, notable positive anomalies of S_n_RF were observed on the landmass near the Arctic Ocean, at altitude on the Rocky Mountains and in the TP regions and negative changes were observed in the mid-latitudes of Eastern Asia and Central United States which is highly identical with spatiotemporal distribution of S_n_RF_m_ displayed in Fig. 4b.

**Figure 5:**
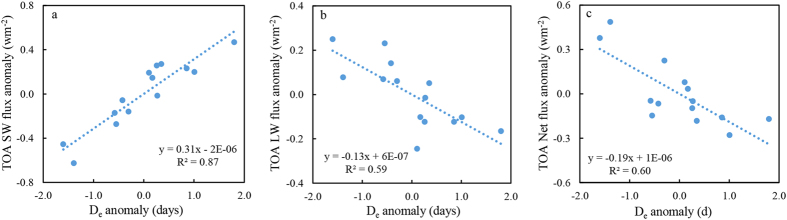
Linear correlation of the snow end date D_e_ anomalies with the TOA (a) SW flux, (b) LW flux and (c) Net flux over the NH snow covered landmass from 2001 to 2013. SW, LW and Net flux were derived from CERES observations. Linear correlations in (**a–c**) are significant at the 95% CL.

Direct estimations using CERES observations further confirmed that changes in D_e_ largely contributed to TOA SW anomalies (Fig. 5a and Supplementary Fig. S13) as suggested by the correlation coefficient of 0.87 between D_e_ and the TOA SW flux anomaly during the melting season, from 2001 to 2013. Changes in D_e_ influenced the SW flux more than the longwave (LW) flux (Fig. 5b). This resulted in the positive anomaly of TOA net flux and led to further warming. The D_e_-induced S_n_RF_m_ of 0.31 (±0.01) Wm^−2^ accounted for 51% of the total TOA SW flux anomaly (0.61 Wm^−2^) and 63% of the TOA Net flux changes (0.50 Wm^−2^) in melting season over the NH snow-covered landmass from 2001 to 2013. In addition, the observed TOA SW flux changes are highly consistent with the observed D_e_ anomalies from mid-to-high latitudes, as a weakened cooling effect is observed in the high latitudes as well as an enhanced cooling effect in the mid-latitudes, which indicates that changes in D_e_ may further influence and control local and regional climate change.

NH landmass warming from 2001 to 2013 was estimated from the Goddard Institute for Space Studies (GISS) Surface Temperature Analysis^40^ at 0.07 °C. Combining the amplitudes of S_n_RF, S_n_RF_a_ and S_n_RF_m_ with this warming estimate (ΔT) yields a NH snow albedo feedback (S_n_RF/ΔT) of 2.29 (±0.06), 0.14 (±0.01) and 4.43 (±0.14) Wm^−2^K^−1^, respectively. The NH Cryosphere radiative forcing caused by snow cover variability between 1979 and 2008^6^ was estimated at 0.27 (0.11–0.48) Wm^−2^ with a warming of 0.79 °C from GISS Surface Temperature Analysis, which yields a snow albedo feedback of 0.34 (0.14–0.61) Wm^−2^K^−1^. Compared with results from Flanner *et al.*^6^, our results indicate that even though the NH snow radiative forcing S_n_RF from 2001 to 2013 (0.16 Wm^−2^) was about 60% of S_n_RF from 1979 to 2008 (0.27 Wm^−2^ Wm^−2^), the NH snow albedo feedback during 2001–2013 (2.29 Wm^−2^K^−1^) was about six times of those from 1979 to 2008 (0.34 Wm^−2^K^−1^) due to a lower warming extent.

Our results based on the snow cover phenology matrix obtained from several datasets from 2001 to 2014 indicate contrasting snow cover phenology characteristics in the NH middle and high latitudes. These results can help to better understand the spatial and temporal changes of snow cover phenology at a continental scale, in the context of the recent climate change, and help to predict it. Climate models made at a continentental scale should take the contrasting features of snow cover phenology into consideration to reduce uncertainties in climate projections. However, the availability of long, high-resolution time-series of snow cover observations from satellites is expected to improve the future study of snow cover phenology. The warming trend is very likely to continue with the accumulation of greenhouse gas in the atmosphere; as well as the intensified effects of the Arctic Amplification resulting from the vanishing cryosphere snow and sea ice. Accordingly, the contrasting changes in the snow cover phenology are very likely to continue in the near future. Moreover, snow cover phenology is largely determined by temperature and precipitation anomalies. According to the similarities between the distribution of snow radiative forcing, the observed TOA SW flux anomalies and the observed contrasting anomalies of D_e_ over the NH, it is necessary to further investigate how these energy budget anomalies control climate change.

## Methods

### Study area

In order to discern variations in the NH snow cover phenology, the snow cover regions (grid cells) was defined as the area ranging between 32°N and 75°N, excluding grid cells of permanent snow cover. The northernmost part of Greenland was excluded from the analysis as there are few products providing reliable snow cover information due to the complex coastal topography and the difficulty in discriminating snow from ice^5^. The region below 32°N was also excluded from the analysis as patchy snow or thin snow covering vegetated surfaces may be missed in the Normalized Difference Snow Index (NDSI) calculated using an algorithm for snow mapping that processes visible and near-infrared images, such as MODIS^41^. In addition, snow can melt and reappear over the course of the snow season. At higher latitudes or altitude, this may only occur at the beginning or at the end of the season, whereas at lower latitudes this may occur throughout the winter^14^. To allow for inter-annual comparisons of snow cover phenology, this study is restricted to stable snow-covered regions where snow covered the ground for at least 60 days each year from 2001 to 2014.

### Snow cover phenology retrieval

Five snow datasets derived from satellite observation and reanalyzes are analyzed (Table S1) in this study. For daily CMC snow depth observations, D_o_ is defined as the first five consecutive days and D_e_ is defined as the last five consecutive days when snow depth exceeds 1 cm, over a given year (t). For the daily IMS binary SCE mask dataset, D_o_ and D_e_ are defined as the first and last five consecutive images when pixels were marked as 1 in the records, over a given year (t). For the daily NISE SWE dataset, D_o_ and D_e_ are defined as the first and last five consecutive days when the snow depth exceeds 2.5 cm, over a given year (t). For the weekly NHSCE binary SCE mask dataset, we first identified the date range (i to i + 6) of the first frame (n) when snow cover occurred and disappeared, respectively. Then, over a given year (t), D_o_ and D_e_ are defined at i + 3, relative to the first frame when snow cover occurred and disappeared, respectively. For the 8-day MOD10C2 snow cover fraction dataset, we first identified the interval (i to i + 7) limits between the frames when the snow cover fraction is superior to 0 and subsequently equals 0, then defined D_o_ and D_e_ at i + 3.5 days of first frame when the snow cover fraction is superior to 0, and equal to 0, respectively.

In order to keep all the information held in the five individual datasets as well as to establish spatial comparisons and causal analyses between snow phenology information obtained from each one of them, we first identify D_o_, D_e_ and D_d_ from individual datasets without regridding. Then, the results obtained from individual datasets are regridded at 0.5 degree spatial resolution using a resampling method of “average” with the help of gdalwarp (http://www.gdal.org/gdalwarp.html). The applied resampling method computes the average of all non-NODATA contributing pixels in the domain of our study.

### Multi-data snow cover phenology retrieval and uncertainty analysis

A multi-data approach^5^ is employed to develop an integrated snow cover phenology matrix and reduce the uncertainty from individual datasets. Taking D_e_ as an example, individual datasets of snow observations suggest different values and spatiotemporal distributions of D_e_ (Supplementary Figs S1 and S2, supplementary Tables S2 and S3) depending on the spatial resolution, the method (or algorithm) used to detect snow cover, and the different definitions of snow cover. In order to achieve internal consistency, D_e_ estimates derived from each individual dataset were converted to standardized z-score anomalies using the mean and standard deviation values observed over a given period (Supplementary Fig. S4). The consistency of each dataset was evaluated by computing the correlation and the root-mean-square error (RMSE) of the multi-dataset mean, excluding the dataset being verified (Supplementary Tables S4 and S5). The final integrated multi-dataset D_e_ series was obtained by averaging the anomaly series from 2001 to 2014. The final D_e_ series was then converted back to a D_e_ value for each day of the year using the mean and standard deviation obtained from the MODIS dataset since MOD10C2 constitutes a source of consistent and objective snow cover phenology estimates, derived from high resolution optical satellite data, compared with the CMC, NHSCE, IMS and NISE snow datasets used in this study. This approach was also applied at pixel level to help remove the individual D_e_ series with poor data quality from the final averaged anomaly series. The correlation and RMSE of each D_e_ anomaly series with the average anomaly series from the other four snow data sets are displayed in supplementary Fig. S5. An estimate of the uncertainty in D_e_ is obtained from the standard error (SE),


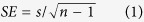


which depends on the standard deviation s of the n data sets included in the average anomaly. The multi-data D_o_ and D_d_ series were estimated following the same process.

For interannual and spatiotemporal anomalies in D_o_, D_e_ and D_d_, we first calculated interannual and spatiotemporal changes from each individual dataset. Then, averaged the individual changes with significance above 90% CL to obtain the final multi-data anomalies. The estimate of the uncertainty in the final multi-data changes is obtained from SE (equation 1), which depends on the standard deviation s of the n data sets included in the final multi-data changes calculation. In addition, we apply 2 s control limits (−2 and +2 times standard error) in the calculation of multi-data anomalies. Individual anomalies beyond this range were also excluded in this study.

### Snow radiative forcing (S_n_RF) calculation using albedo radiative kernels

The albedo radiative kernel is expressed as the TOA net shortwave anomalies associated with a 1% change in the land surface albedo a_s_. In our study, the albedo radiative kernel — estimated using radiative transfer algorithms from the Community Atmosphere Model (CAM3) of the National Center for Atmosphere Research as well as the Atmosphere Model 2 (AM2) of the Geographical Fluid Dynamic Laboratory, developed by Shell *et al.*^38^ and Soden *et al.*^37^— is used to quantify the S_n_RF. We define the S_n_RF as the instantaneous perturbation to Earth’s TOA SW anomalies consequent of the snow season variability, which can be quantified as a_s_ anomalies driven by the disappearance of snow cover during the melting season and by the presence of snow cover during the accumulation season. Thus, the time (t) dependent S_n_RF within the study area of area A, which is composed of gridcells r, can be represented as follows:


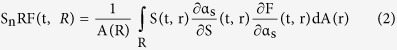


Here, S is the snow cover fraction over the study area. ∂α_s_/∂S is the rate of variation in surface albedo with the snow cover changes. ∂F/∂α_s_ is the response of TOA SW flux variation to a_s_ changes. We assume that (monthly and spatially varying) ∂α_s_/∂S and ∂F/∂α_s_ are constant with snow cover fraction S and surface albedo α_s_, respectively. Then, ∂α_s_/∂S can be replaced with mean albedo contrast induced by snow cover anomaly and ∂F/∂α_s_ can be obtained from the albedo radiative kernels^6^,^39^.

## Additional Information

**How to cite this article**: Chen, X. *et al.* Observed contrast changes in snow cover phenology in northern middle and high latitudes from 2001–2014. *Sci. Rep.* **5**, 16820; doi: 10.1038/srep16820 (2015).

## Supplementary Material

Supplementary Information
