# Supplementary material for: Observed contrast changes in snow cover phenology in northern middle and high latitudes from 2001–2014
Source: Sci Rep. 2015 Nov 19;5:16820. doi: 10.1038/srep16820 (PMC4652201; doi:10.1038/srep16820)
Supplement: Supplementary Information [file srep16820-s1.pdf]

Supplementary Information

Observed contrast changes in snow cover phenology in northern middle and high latitudes from 2001-2014

Xiaona Chen[1, 2], Shunlin Liang[1, 2, *], Yunfeng Cao[1, 2], Tao He[2], Dongdong Wang[2]

[1]State Key Laboratory of Remote Sensing Science, School of Geography, Beijing Normal University, Beijing 100875, China

[2]Department of Geographical Sciences, University of Maryland, College Park 20742, USA

Correspondence to: sliang@umd.edu

Text S1: Background and importance of snow cover phenology study

Snow cover over the Northern Hemisphere (NH) plays a crucial role in the Earth's hydrological cycle and in energy balance its surface. It modulates feedbacks that control variations of the global climate [S1]. Previous studies have proven that the snow cover in the NH has experienced a well-documented rapid decrease [S2-5]. For example, based on an estimation obtained from multiple datasets, Brown, et al. [S5] found that the May and June snow cover extents (SCE) have decreased by14% and 46%, over the pan-Arctic region from 1967 to 2008. Derksen and Brown [S3] further proved that the June SCE had decreased nearly twice as fast as the widely publicized September sea ice extent did between 1979 and 2011, especially in the later part of that period. SCE reductions in the 2008–2012 period have even exceeded climate model projections [S3].

This trend will continue in the future according to climate projections[S4,S6], coincident Changes in SCE coincide with hemispheric warming and are indicative of a positive feedback of surface reflectivity on climate [S4,S5,S7,S8]. With SCE retreats, less solar radiation was reflected to space, which may resulted in additional absorbed solar radiation by their Earth system. Flanner, et al. [S7] estimated a mean forcing of −2.9 to −1.2 Wm^{-2} in the NH caused by snow cover reduction. Cryospheric cooling declined by 0.45 Wm^{-2} from 1979 to 2008, with nearly half of that value being attributed to changes in land snow cover [S7]. Fernandes, et al.[S9] found that the snow albedo feedback (SAF) is 1.06 ± 0.08% K^{-1} over the NH snow covered areas with similar magnitudes for the effect of surfaces transitioning from snow covered to snow free conditions and the effect of temperature on surface albedo over snow covered surfaces between 1982–1999.

In response to the rapid decline of SCE, snow cover phenology has experienced remarkable changes, including the shortening of snow cover duration (D_d) period [S10,S11], the earlier melt onset [S12], the earlier snow end date (D_e) and unnoticeable changes in the snow onset date (D_o) [S10] at local and regional scales. For example, Beniston [S13] investigated the variations of snow depth and duration in the Swiss Alps over the 50 years preceding 1997 and concluded that the length of the snow season and the snow amount have substantially decreased; and that large-scale climate forcing plays a dominant role in controlling the timing and amount of snow in the Alps. Choi, et al.[S10] concluded that, in the NH, the average snow season full duration has decreased at a rate of 0.8 week $decade^{-1}$ between the winters of 1972/73 and 2007/08. This is due primarily to a progressively earlier offset advancing at a rate of 5.5 days $decade^{-1}$. There was no significant change in the core snow season in the NH. Wang, et al. [S12] retrieved the pan-Arctic snow melt onset from satellite Passive Microwave (PM) measurements from 1979 to 2011 and found a significant earlier date for the melt onset (2-3 days $decade^{-1}$) concentrated over the Eurasian land sector of the Arctic, which was driven by the spring air temperature at surface level. Peng, et al. [S14] summarized the change in snow phenology and its potential feedback to temperature in the NH over the last three decades using in situ observations and concluded that earlier snow cover termination is systematically correlated on a year-to-year basis with a positive temperature anomaly during the snowmelt month with a sensitivity of −0.077 °C d^{-1}. Mioduszewski, et al. [S15] studied the snowmelt onset in a region of Northern Canada during the spring snowmelt season from 2003 to 2011 and concluded that land cover and the local energy balance may contribute to the variation in the snowmelt onset date. Whetton, et al. [S11] studied the snow cover duration in the Australian Alps and found that even in the best

case scenario for 2030, simulations suggest a decline in average snow cover duration and in the frequency of years of more than 60 days of snow cover, at all sites distributed in the Australian Alps.

However, the changes in snow cover phenology, its attributions and its response to climate change are far away from being well understood since most of the studies above are based on in situ observations, models or on a single source of images. The in situ measurements are highly dependent on the particular locations (latitude and elevation) and limited in spatial coverage. Meanwhile, the use of snow maps generated from a single source may lead to large uncertainties. For example, the visible and near-infrared satellite data are largely influenced by cloud coverage and snow maps derived from PM make it difficult to distinguish wet and shallow snow from wet or snow-free ground according to the assessment of the relative accuracy of hemispheric-scale snow cover maps [S16]. Moreover, the published snow cover phenology mainly focused on the NH high-latitudes, and the hemisphere wide snow cover phenology, and its variations, have not been documented. Thus, there is a need to study the NH snow cover phenology at the continental scale, using the multi-data approach.

Text S2: Summary of datasets

Five snow datasets were used to detect snow cover phenology in our study, including the reanalyzed daily snow depth dataset generated by the Canada Meteorological Center (CMC) [S17], the binary daily snow cover mask derived from the Interactive Multi-sensor Snow and Ice Mapping System (IMS) [S18] and the Northern Hemisphere EASE-Grid 2.0 Weekly Snow Cover and Sea Ice Extent (NHSCE) [S18,S19], 8-Day Level 3 snow cover fraction products (MOD10C2) derived from the Moderate Resolution Imaging Spectroradiometer Satellite (MODIS) [S20] and snow water equivalent (SWE) derived from the Near-real-time Ice and Snow Extent (NISE) dataset [S21].

CMC is generated based on the optimal interpolation of in situ daily snow depth observations with a first-guess field generated from a simple snow accumulation and melt model based on analyzed temperatures and forecasted precipitation from the Canadian forecast model [S17]. Grid cells were considered completely snow covered for snow depth values exceeding 1 cm. Since there are few in situ observations over the Arctic high latitudes, the analysis is based to a large extent on estimated snow depths from the first-guess field. In addition, the available snow depth observations tend to be made in open areas where the snow melts out earlier than in the surrounding terrain [S22].

The binary daily snow cover mask derived from the IMS is manually created by a snow analyst looking at all available satellite imagery, automated snow mapping algorithms, and other ancillary data [S18]. This system relies mainly on visible satellite imagery but also includes station observations and PM data. The IMS daily 24 km snow cover products cover period from early 1997 to the present.

The weekly NHSCE product was generated based on the National Oceanic and Atmospheric Administration (NOAA) /National Climatic Data Center (NCDC) Climate Data Record (CDR) of Northern Hemisphere Snow Cover Extent at a 25km spatial resolution [S19]. The current (version 4) of NHSCE uses an improved algorithm to produce

pseudo-weekly snow charts from the daily, 24 km IMS product after 1997. Compared with IMS, NHSCE comprises the longest satellite-based SCE and covers the period from 4 October 1966 to the present, which makes it possible to discuss the long-term snow cover anomaly. Thus, we use both NHSCE and IMS snow datasets in this study. Moreover, snow cover phenology is highly sensitive to the spatial and temporal resolutions adopted in data sampling, employing both IMS and NHSCE make it possible to resolve and detect effects occurring on a short timescales that contribute to the snow cover phenology. In addition, using NHSCE in this study makes our results comparable to previous studies, such as Choi et al. [S10] and Brown et al. [S5] and Brown and Robinson [S4]. Recent uncertainty analysis indicates an uncertainty in SCE of ± 3–5% at a 95% CL during the NH spring over the 1966–2010 period [S4]. Since the binary value in NHSCE indicates a 50% or greater probability of occurrence of snow in each pixel, this data is most appropriate for large scale snow studies.

The 8-Day snow cover fraction products (MOD10C2) derived from the Moderate Resolution Imaging Spectroradiometer Satellite (MODIS) is created by assembling 8-day composite MOD10A2 products with 500 m spatial resolution which is an eight-day composite of MOD10A1 daily snow cover maps that were derived using a fully-automated algorithm [S20]. The overall absolute accuracy of MOD10A1 is higher than 93% [S23]. The 8-day composite is considered useful because in many regions, particularly at high latitudes, persistent cloudiness limits the number of days available for surface observations [S1]. In this study, the MOD10C2 snow cover dataset was used as a benchmark for other snow observations as MOD10C2 constitute consistent, objective snow estimates derived from high resolution visible satellite data, compared to CMC, NHSCE, IMS and NISE snow datasets used in this study.

The daily SWE derived from the NISE provides global maps of SCE. The SCE mapping algorithm marks a grid cell as snow-covered when a computed snow depth > 2.5 cm in NISE is observed. The NSIDC creates the NISE product using PM data from the Special Sensor Microwave Imager/Sounder (SSMIS) onboard the Defense Meteorological Satellite Program (DMSP) F17 satellite. Previous research has suggested that the maps derived from visible and near-infrared data are more accurate for mapping snow cover than are the PM-derived maps because the difficulty in distinguishing wet and shallow snow from wet or snow-free ground when using PM, according to the assessment on the relative accuracy of hemispheric-scale snow cover maps [S16]. However, recent study proved that although the SWE estimates can be highly uncertain in Arctic regions, standard 19–37 GHz SWE retrievals are able to provide realistic estimates of spring snow extent variability when used only as a proxy for snow cover [S24]. In this study, we only used the proxy SCE information derived from NISE.

One calculated chart date based on the NHSCE and published by Rutgers University Global Snow Lab (http://climate.rutgers.edu/snowcover/) [S25] was used to investigate the long term SCE anomaly. Since monthly SCE is incomplete during 1966-1971, we mainly used SCE from 1972 to 2014 in this study.

The daily ground snow depth measurements generated by the Global Historical Climatology Network (GHCN) version 3.20 [S26] and the European Climate Assessment and Dataset (ECAD) blended version [S27] were used to identify the in situ observed snow cover phenology. By assembling and checking observations made in many different nations, both

the GHCN and ECAD datasets contain daily snow depth records over the NH snow covered landmass, especially in high latitudes.

The long-term, high-quality, and spatially complete Global LAnd Surface Satellite (GLASS) albedo product [S28,S29] was used to identify the changes in land surface albedo and calculate the albedo contrast caused by snow cover phenology changes with the help of the MODIS-derived global gap-filled snow-free land surface albedo product (MCD43GF) [S30]. The GLASS land surface albedo dataset was produced from both AVHRR and MODIS data and has been used to quantify the radiative forcing of snow melting over Greenland [S31] with high quality and fine spatial resolution, as well as global land surface albedo climatology [S32]. The MCD43GF were employed to get the climatological value of spatially complete snow-free land surface albedo, which is used to calculate the snow radiative forcing (S_nRF) induced by land surface albedo changes at the top of atmosphere (TOA). The spatially complete albedo is produced using an ecosystem-reliant temporal interpolation technique that retrieves missing data with 3–8% error [S30]. Prior to calculation, both the GLASS and the MCD43GF surface albedo values were reproduced to generate the monthly mean value. During the process of reproduction, the selected quality control (QC) flags with “00” and “01”, indicating uncertainty of <5 and 10%, respectively, were used to generate the monthly mean value of GLASS. Quality assurance values between 0 and 1, representing overall high-quality full inversion values, were used to generate the climatology value of monthly MCD43GF snow-free land surface albedo.

The Clouds and Earth’s Radiant Energy System (CERES) satellite products [S33], including global measurements of broadband (0.2-5 µm) shortwave (SW) reflected flux, broadband (5-100 µm) Longwave (LW) reflected flux and the broadband (0.2-100 µm) incoming solar minus the reflected SW and LW emitted (Net) flux at the TOA, were used to evaluate the influence of snow cover phenology change on the Earth’s climate system. In order to eliminate the role of cloud feedback, we employed in our study the variables observed in clear-sky conditions derived from CERES Energy Balanced and Filled (EBAF) products. The EBAF clear-sky filled product is spatially complete, as clear-sky fluxes are inferred from both CERES and MODIS measurements to produce a new clear-sky TOA flux climatology that provides TOA fluxes in each 1˚x1˚ region every month [S34].

Monthly surface temperature and precipitation derived from the Climatic Research Unit (CRU) Time-Series (TS) Version 3.22 [S35] were used to quantify the causes of snow phenology changes. The CRU dataset is gridded at a 0.5° spatial resolution based on the analysis of over 4000 individual weather station records.

The radiative kernel expressed as the TOA net shortwave anomalies associated with a 1% change in the surface albedo estimated using radiative transfer algorithms from the Community Atmosphere model (CAM3) in the National Center for Atmosphere Research (NCAR) and the Atmosphere Model 2 (AM2) of the Geographical Fluid Dynamic Laboratory developed by Shell et al. [S36] and Soden et al. [S37] were used to quantify the snow radiative forcing caused by the land surface albedo change in response to snow cover anomaly.

In order to keep the snow information in five different snow datasets and study the spatial characteristics of snow cover phenology as well as to establish the mechanisms controlling

change, we first identify the snow cover phenology based on different snow datasets. Then, all the results were converted to gridded D_o, D_e and D_d at a 0.5-degree spatial resolution with the help of gdalwarp (http://www.gdal.org/gdalwarp.html). The average resampling computes the average of all non-NODATA contributing pixels in our study.

Text S3: Multi-data snow cover phenology retrieve and uncertainty analysis

Taking the snow end date D_e as an example as displayed in Fig.S2 and Fig.S3, the individual sources of snow observations differ in mean amplitude and spatial distribution depending on spatial resolution, the method (or algorithm) used to detect snow cover, as well as different definitions of snow cover. This disparity in D_e retrieved from individual snow observations mainly distributed over high elevation regions in the mid-latitudes, especially over the Tibet Plateau (TP) and the Rocky Mountains area, in which D_e derived from CMC is much later than the value derived from the other four observations, especially NHSCE. Meanwhile, D_e derived from NISE is much earlier than the value derived from the other four data sets. This is mainly caused by the different definitions of snow cover used by CMC and NISE and described above. A summary of the snow onset date (D_o), D_e and the snow duration days (D_d) estimates from the five snow datasets from 2001 to 2014 (Table S2) show that the D_e averages around 127.86 (day of year) with an inter-dataset standard deviation of 5.61 days. The minimum D_e derived from NISE is of 104.62 and maximum D_e retrieved from MODIS is found to be 135.67. 14-year changes of D_e calculated by linear regression model for each dataset displayed earlier D_e in almost all data sets, however, the changes in CMC and NISE are not statistically significant at 95% confidence level (CL) as described in Table S3.

In order to compare the datasets on a consistent basis, D_e derived from different datasets were converted to standardized anomalies using the mean and standard deviation calculated for the 2001-2014 period. The impact of converting the individual D_e series into a series of standardized z-score anomaly is demonstrated in Figs.S4c and S4d. The consistency of each dataset was evaluated by computing the correlation and root mean square error (RMSE) between each dataset and the multi-dataset mean excluding the data set being verified.

The evaluation of the consistency of D_e is summarized in Tables S4 and S5. The results indicate generally consistent agreement between D_e series derived from each individual dataset. In most cases, correlation coefficients are greater than 0.6 and RMSE less than 0.84. CMC and NHSCE derived D_e series are exceptions with notable discrepancies from the multi-data set average, due to snow identification issues mentioned above. The multi-dataset average was observed to be in consistently good agreement with MODIS benchmarks, with a highest correlation coefficient of 0.93 and a lowest RMSE of 0.37. The performance of NISE is better than CMC and NHSCE in D_e identification with a correlation of 0.71 and a RMSE of 0.72, which means that the added microwave-based NISE observations provide additional justification for using a multi-dataset approach for mapping D_e over the NH.

It had been intended to remove the poorer performing D_e series from the final average anomaly series. However, multiple regression analysis revealed that all of the datasets were statistically significant (95% CI) variables in explaining the variance in the multi-dataset anomaly series so there was no compelling reason to eliminate any of the datasets from the final anomaly series. This approach was also applied at pixel scale to remove the poorer performing individual D_e series from the final average anomaly series in each pixel. The correlation and RMSE of D_e anomaly series with the average anomaly series from the four other data sets above the 95% CI, over the NH from 2001 to 2014, are displayed in Fig.S5.

The stratification of the evaluation results in Tables S4 and S5, referring to the correlation and RMSE analysis, reveal that the highest resolution dataset (MOD10C2) has the highest correlation and lowest RMSE compared to the multi-data set average. Among the medium resolution datasets (IMS, CMC, NISE and NHSCE), the visible and infrared data based IMS dataset with daily temporal resolution performs better, followed by CMC and NISE. NHSCE shows the poorest performance among medium resolution datasets due to issues related to the adopted definition of snow and to the temporal resolution.

Text S4: Validating multi-data snow cover phenology using in situ observations

In situ snow depth data are used to verify the performance of the multi-data set snow cover phenology to capture the distribution of "real" snow cover phenology in our study. To avoid the impact of ephemeral snow on the ground on the identification of snow onset date D_o, snow end date D_e, and snow duration days D_d as well as match the temporal resolution of satellite observations, for GHCN and ECAD daily ground snow depth measurements, D_o is defined as the first consecutive five days in the accumulation season and D_e is defined as the last consecutive five days of persistent snow cover in the melting season. D_d is defined as the number of days from the onset of snow cover to the end of snow cover. Limited by the data availability of GHCN and ECAD, in situ observed D_o, D_e, and D_d from 2001 to 2012 were used in the comparison.

Fig.S6 shows the spatial patterns of the 12-year averaged D_o, D_e, and D_d over the GHCN and ECAD covered stations during 2001–2012. There are clear latitudinal gradient patterns for D_o, D_e, and D_d from middle to high latitudes. In most sites, the observed D_o, D_e, and D_d are consistent with the multi-data retrieved D_o, D_e, and D_d results, R^2 varies as 0.72, 0.76, and 0.81 at the 95% CI. However, compared with the bias in D_o and D_e, the bias in observed D_d and the multi-data retrieved D_d is much larger. This is mainly because the bias in D_d superimposed the bias in D_o and D_e. Moreover, the value of multi-data retrieved snow cover phenology are higher than the in situ observed snow cover phenology in low D_o, D_e, and D_d value distributed regions with opposite trends in high D_o, D_e, and D_d value-distributed regions as demonstrated in Figs.S6g, S6h, and S6i. This phenomena was mainly caused by the low spatial resolution of the multi-data snow cover phenology series, which provides the average D_o, D_e, and D_d value in pixel scale. Limited by spatial resolution of the snow date sets used in this study, the minima unit in the multi-dataset snow cover phenology are pixels in 0.5°, which cannot totally catch and reflect the "real" value of D_o, D_e, and D_d specific spot location.

The error distribution of differences between the in situ observed D_o, D_e, and D_d, and multi-data retrieved D_o, D_e, and D_d (in situ observed D_e minus multi-data retrieved D_e) are shown in Figs.S6j, S6k, and S6l. The performance of satellite-retrieved D_o, D_e, and D_d with low elevation are better than its performance with high elevation. The stations with elevation <500 m, 500–1000 m, and >1000 m account for 77.3%, 12.5%, and 10.2% of the total 216 stations used in this study, in which 86% and 69% of the stations at an elevation >1000 m record an error range beyond -5 days to 5 days in D_e and D_d, respectively.

Previous research has proved that the raw in situ observations would give results that are highly dependent on the particular locations (latitude and elevation) and reporting periods of the actual weather stations. Such results would mostly reflect those accidental circumstances rather than yield meaningful information about the climate[S38]. However, as shown in Fig.S6, the multi-dataset D_o, D_e, and D_d still skillfully captures D_o, D_e, and D_d over the NH when the bias is -0.89, -1.96, and -6.32 days as compared to the in situ observed results, which proves that the multi-data approach used in our study is reliable.

Text S5: Sensitivity and Contribution Calculation

Snow cover phenology is largely determined by the land surface temperature and precipitation data. For sensitivity of D_o to T_a and P_a, we regressed D_o as the dependent variable with T_a and P_a as independent variables.

$$D_o = a\times T_a + b\times P_a + c \tag{1}$$

Here, we assumed that the interannual variability of D_o was driven by T_a and P_a, which is reflected by the term $a\times T_a$, $b\times P_a$. Then, we defined the regression coefficient a as the sensitivity of D_o to T_a, which removed the effects of P_a on D_o, and the regression coefficient b as the sensitivity of D_o to P_a, which removed the effects of T_a on D_o. This formula was employed by Peng, et al. [S14] to identify the sensitivity of D_e to the temperature.

D_e mainly depends on T_m and the maximum spring snow depth to melt, in which the maximum snow depth in spring is determined using T_a and P_a[S14,S39]. For the sensitivity of D_e to T_a, P_a and T_m, we performed a multiple linear regression using D_e as the dependent variable with T_a, P_a, and T_m as independent variables.

$$D_e = d\times T_a + e\times P_a + f\times T_m + g \tag{2}$$

Here, we assumed that interannual variations of D_e come from changes in T_a, P_a, and T_m, which are reflected by the term $d\times T_a$, $e\times P_a$ and $f\times T_m$. Then, the regression coefficient d is defined as the sensitivity of D_e to T_a, which removed the effects of P_a and T_m on D_e, the regression coefficient e as the sensitivity of D_e to P_a, which removed the effects of T_a and T_m on D_e, and the regression coefficient f as the sensitivity of D_e to T_m, which removed the effects of T_a and P_a on D_e.

D_d is largely decided by the entire snow season temperature (T_s) and accumulated snow depth, which is the resultant of T_a, T_m, and P_a. For sensitivity of D_d to T_s and P_a, we regressed D_d as the dependent variable with T_s and P_a as the independent variables.

$$D_d = h\times T_s + i\times P_a + j \tag{3}$$

Here, we assumed that the interannual variations of D_d comes from changes in T_s, P_a, which is reflected by the term h× T_s, i× P_a. Then, the regression coefficient h is defined as the sensitivity of D_d to T_s that removed the effects of P_a on D_d and the regression coefficient i is defined as the sensitivity of D_d to P_a which removed the effects of T_s on D_d.

To compare the contributions of D_o, D_e, and D_d from each variable consistently, all the variables were converted to the standardized anomalies (z-score) using the mean and standard deviation in the contribution calculation. Contributions of each variable to D_o, D_e, and D_d variability were computed annually and zonally to display the changing influence of accumulation and melting season climate on the underlying snow cover phenology variability. The contributions of T_a and P_a to D_o were computed by regressing the annual and zonal time series of D_o z-scores against time series of z-scores of T_a and P_a. The resulting regression coefficients were then multiplied by the time series of T_a and P_a z-scores to derive the contributions of T_a and P_a to the D_o z scores. Similarly, the contributions of T_a, P_a, and T_m to D_e were computed by regressing the annual and zonal time series of D_e z-scores against the time series of z-scores of T_a, P_a, and T_m. The resulting regression coefficients were multiplied by the time series of T_a, P_a, and T_m z-scores to derive the contributions of T_a, P_a, and T_m to the D_e z-scores. The contributions of T_s and P_a to D_d were computed by regressing the annual and zonal time series of D_d z-scores against time series of z-scores of T_s and P_a. The resulting regression coefficients were then multiplied by the time series of T_s and P_a z-scores to derive the contributions of T_s and P_a to the D_d z-scores.

Supplementary Figures

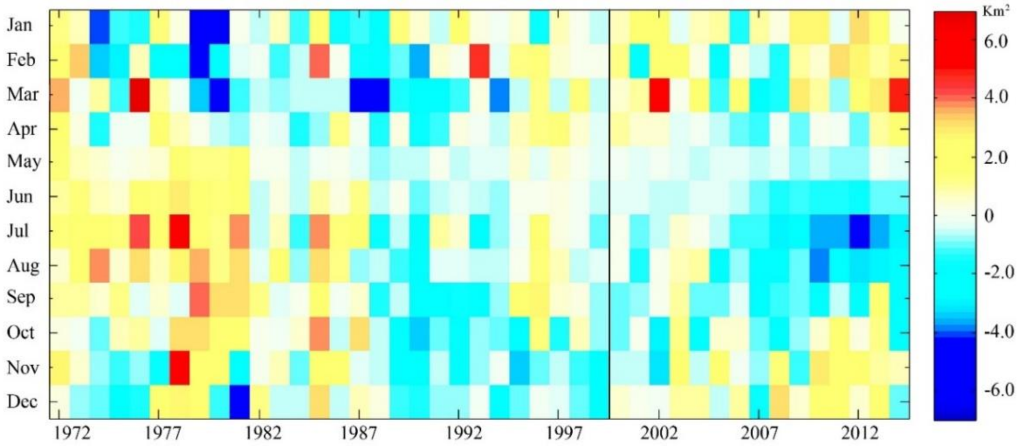

Figure S1. Monthly snow cover extent (SCE) anomaly from 1972 to 2014 over the NH. Chart data was calculated based on NH SCE CDR v01r01[S25] and published by the Rutgers University Global Snow Lab (http://climate.rutgers.edu/snowcover/). The anomaly value was calculated by monthly SCE minus 43-year averaged SCE in corresponding month.

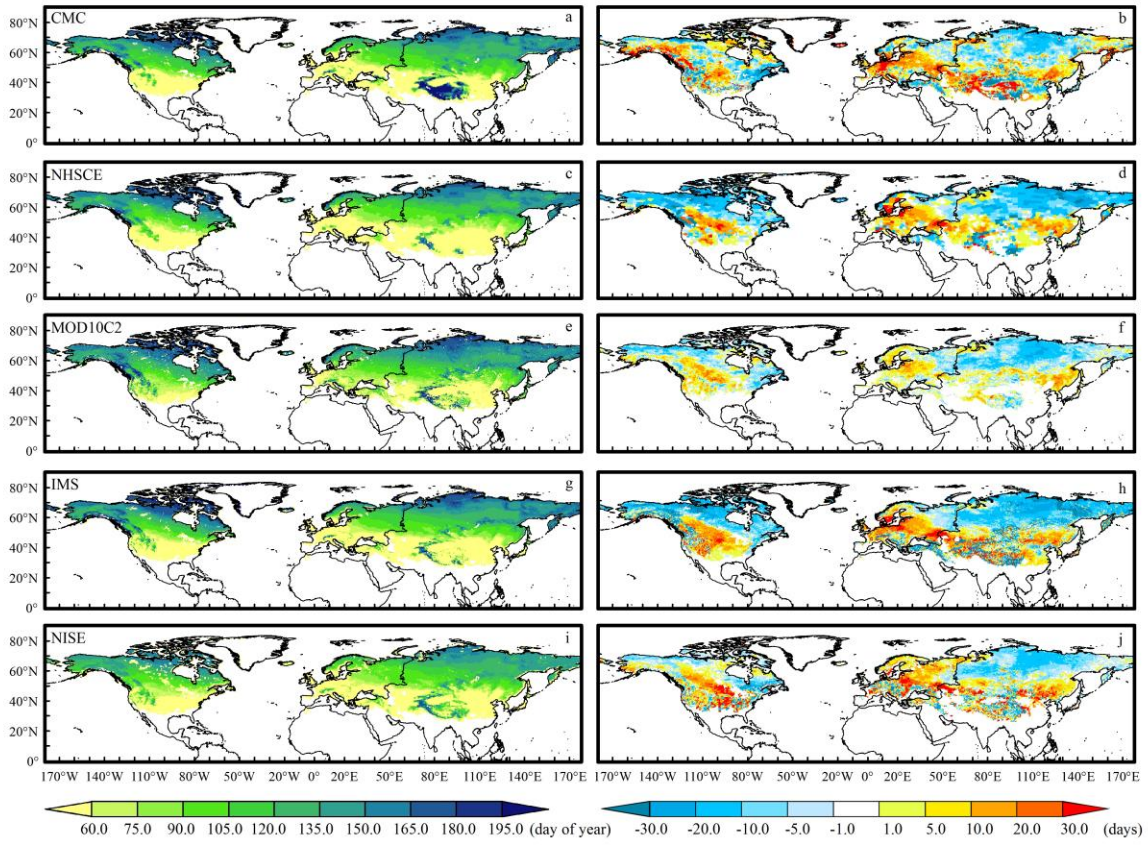

Figure S2. 14-year averaged values of snow end date (D_e) in day of year detected by (**a**) CMC, (**c**) NHSCE, (**e**) MOD10C2, (**g**) IMS, and (**i**) NISE from 2001 to 2014. 14-year changes of D_e calculated from linear regression model from CMC, NHSCE, MOD10C2, IMS, and NISE are presented in (**b)**, (**d**), (**f**), (**h)**, and (**j**). Changes are derived from linear slope are multiplied by the time span. The figure was created using *ArcGIS* (version10.2.2) [S40].

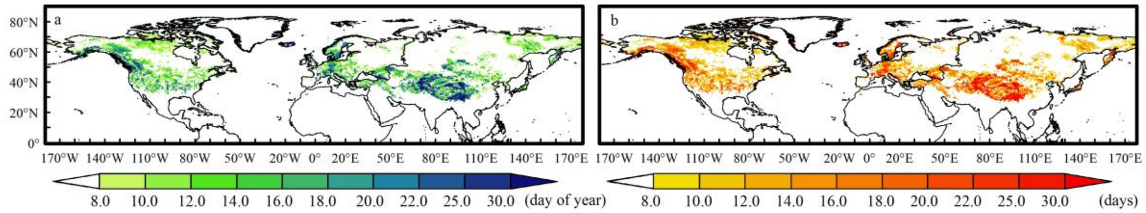

Figure S3. Standard deviation of 14-year (**a**) averaged D_e and (**b**) changes detected by five individual snow datasets summarized in **Table S1** from 2001 to 2014. The figure was created using *ArcGIS* (version10.2.2) [S40].

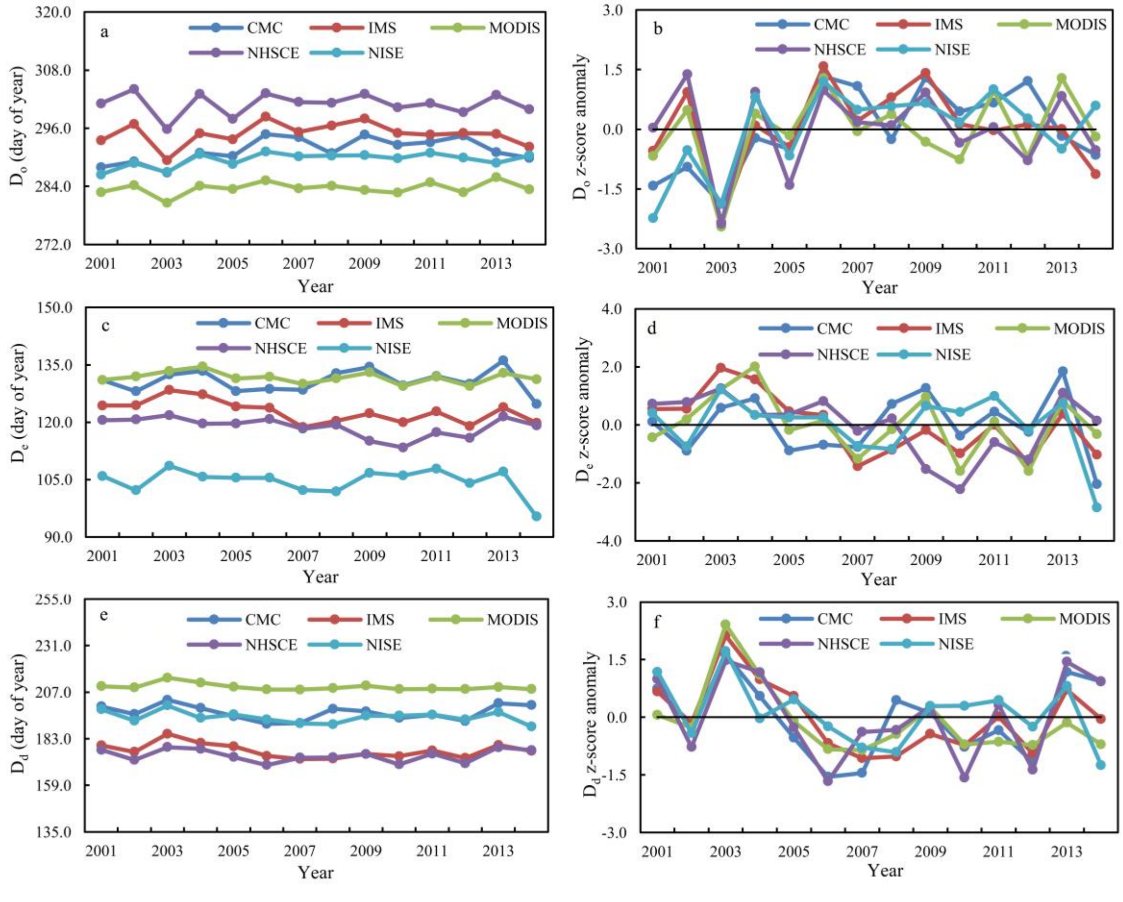

Figure S4. Annual averaged raw (**a**) D_o, (**c**) D_e, and (**e**) D_d series and the corresponding standardized (**b**) D_o, (**d**) D_e, and (**f**) D_d anaomaly (z-score) series detected by five snow data sets over the NH from 2001 to 2014.

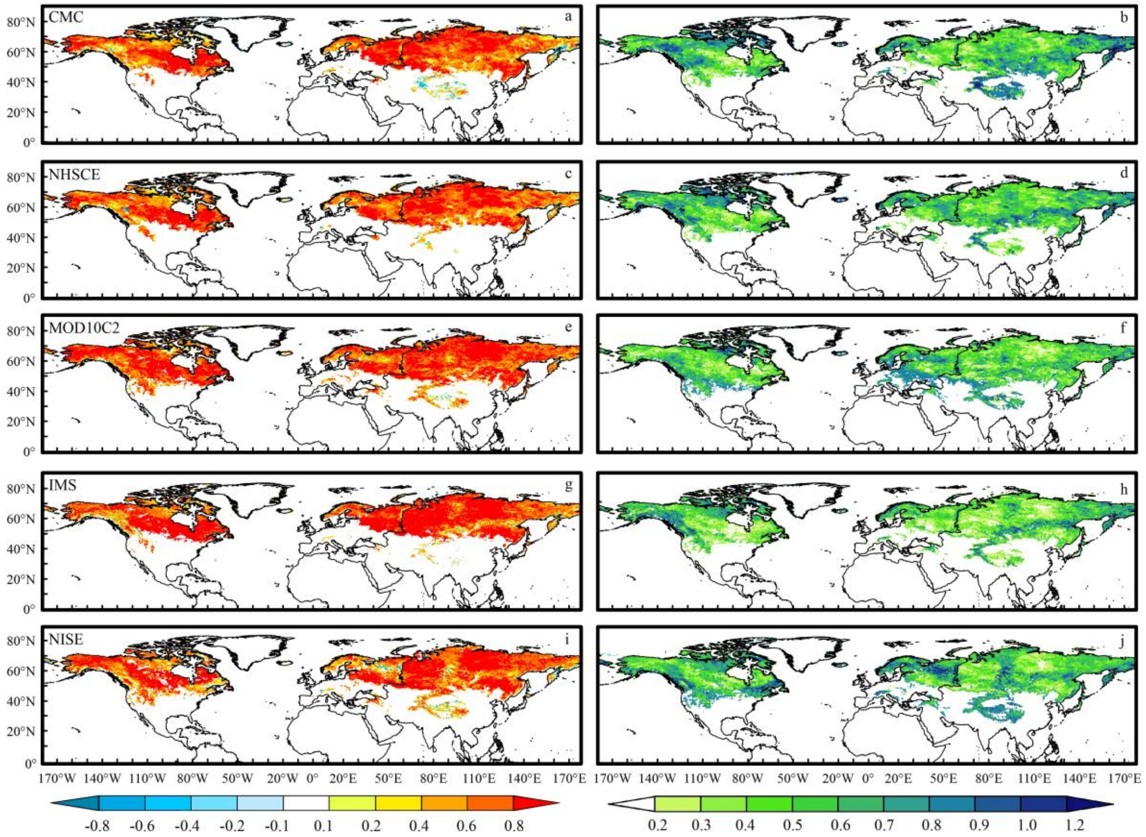

Figure S5. Correlation and RMSE of each D_e anomaly (z-score) series with the averaged anomaly series from the other four snow data sets used in this study. Left column is the correlation of (**a**) CMC, (**c**) NHSCE, (**e**) MOD10C2, (**g**) IMS, and (**i**) NISE with the average anomaly series from the other four snow datasets. Right column is the corresponding RMSE value of (**b**) CMC, (**d**) NHSCE, (**f**) MOD10C2, (**h**) IMS, and (**j**) NISE with the average anomaly series from the other four snow data sets. The figure was created using *ArcGIS* (version10.2.2) [S40].

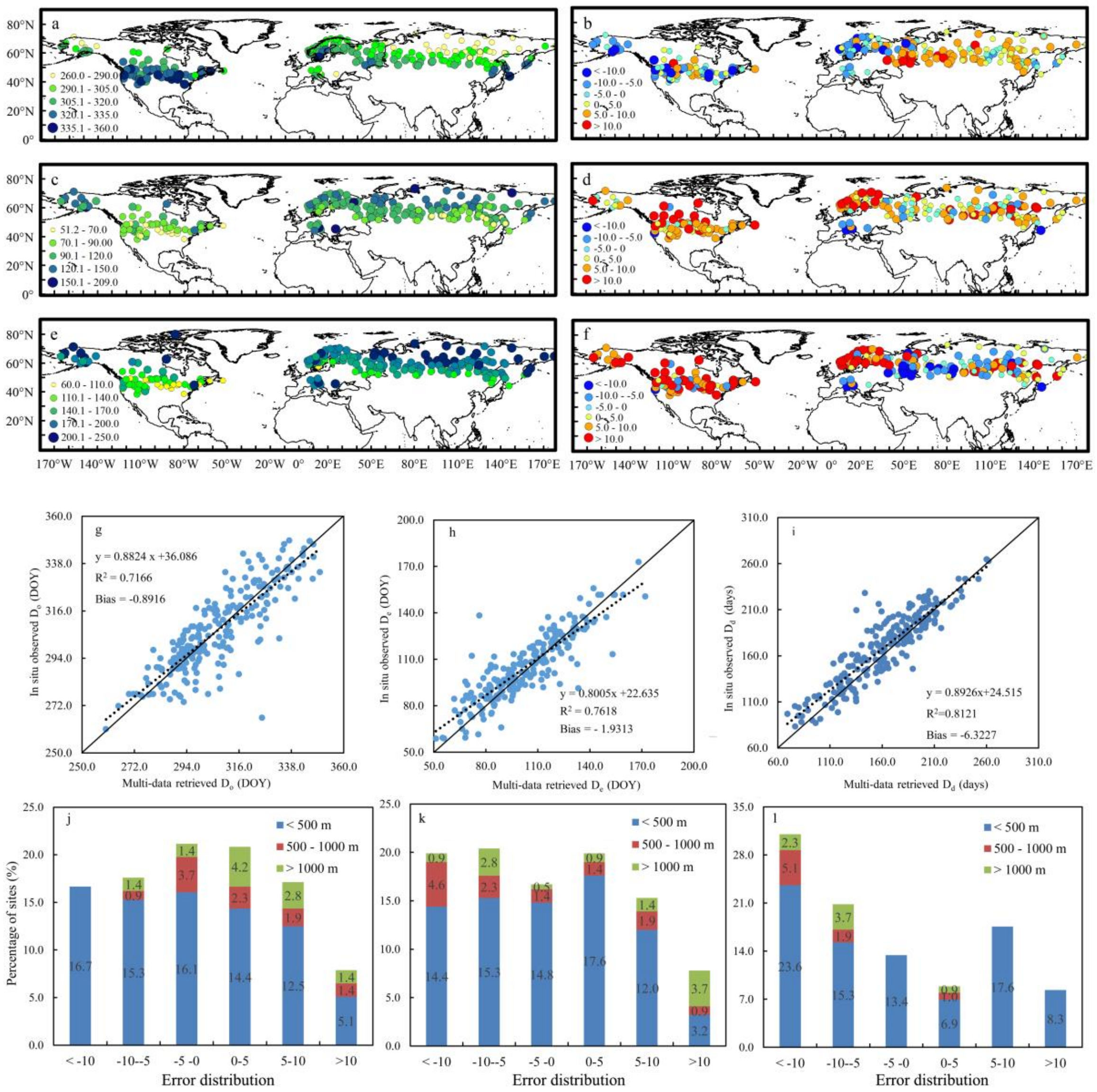

Figure S6. 12-year annual averaged value of (**a**) D_o, (**b**) D_e, and (**c**) D_d derived from the GHCN and the ECAD in situ snow observations from 2001 to 2012. (**d**) Elevation distributions of in situ snow observations from GHCN and ECAD. Scatter plots of 12-year averaged in situ observed and multi-dataset retrieved (**e**) D_o, (**f**) D_e, and (**g**) D_d. We employed data from 2001 to 2012 instead of 2001 to 2014 because of unavailability of in situ snow depth in 2013 and 2014 in most of GHCN and ECAD stations. The figure was created using *ArcGIS* (version10.2.2) [S40].

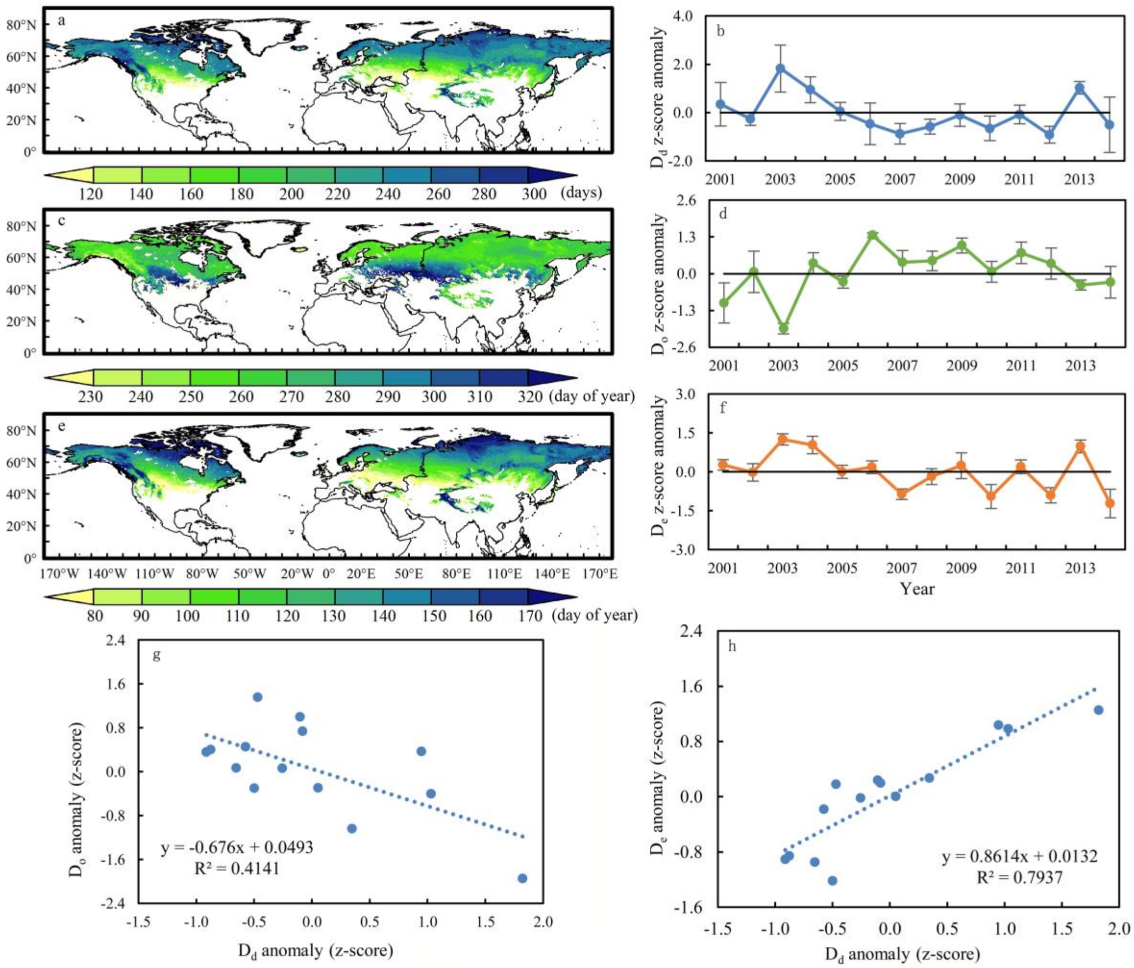

Figure S7. Spatial distribution of 14-year averaged value of (**a**) D_d, (**c**) D_o, and (**e**) D_e retrieved from multi-data series from 2001 to 2014. 14-year anomaly (z-score) of (**b**) D_d, (**d**) D_o, and (**f**) D_e from 2001 to 2014. (**g**) Correlation between D_o and D_d anomaly series. (**h**) Correlation between D_e and D_d anomaly series. Error bar in (**b**), (**d**), and (**f**) are generated from standard error in multi-data D_d, D_o, and D_e series. The figure was created using *ArcGIS* (version10.2.2) [S40].

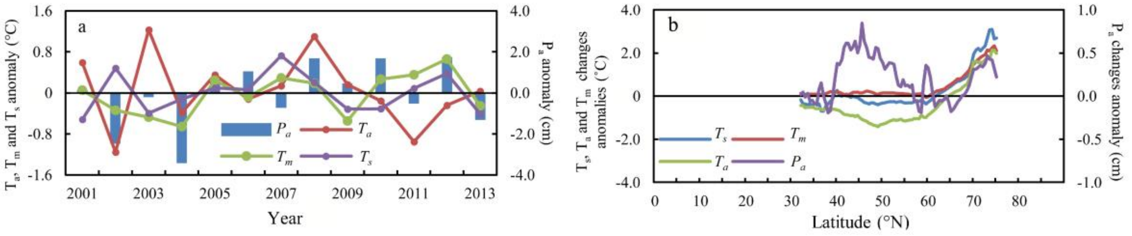

Figure S8. (**a**) Annual-averaged T_a, T_m, T_s, and P_a anomalies from 2001 to 2013. (**b**) Zonal averaged T_s, T_a, T_m, and P_a anomalies from 2001 to 2013.

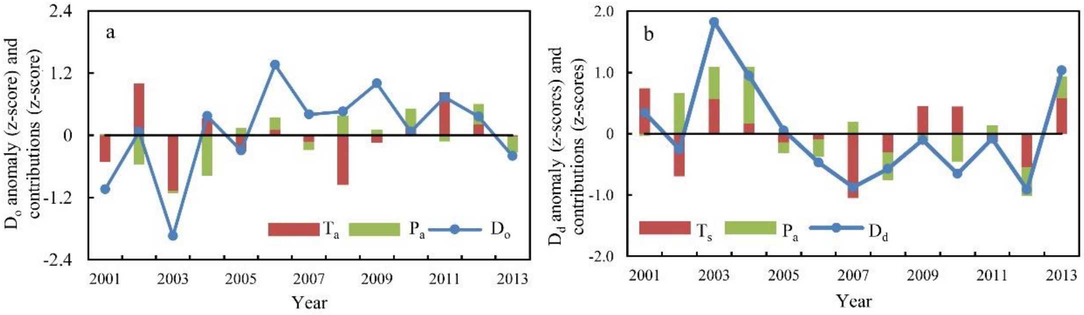

Figure S9. (**a**) Annual averaged D_o anomaly (z-score) and contributions (z-score) from T_a and P_a. (**b**) Annual averaged D_d anomaly (z-score) and contributions (z-score) from T_s and P_a.

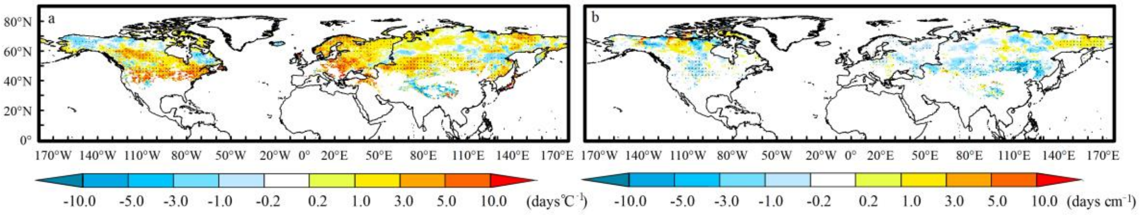

Figure S10. (**a**) Sensitivity of D_o to T_a when the impacts of P_a are removed. (**b**) Sensitivity of D_o to P_a when impacts of T_a are removed. Black dots indicate correlation between D_o and T_a in (**a**), D_o and P_a in (**b**) are significant at 90% CL. The figure was created using *ArcGIS* (version10.2.2)[S40].

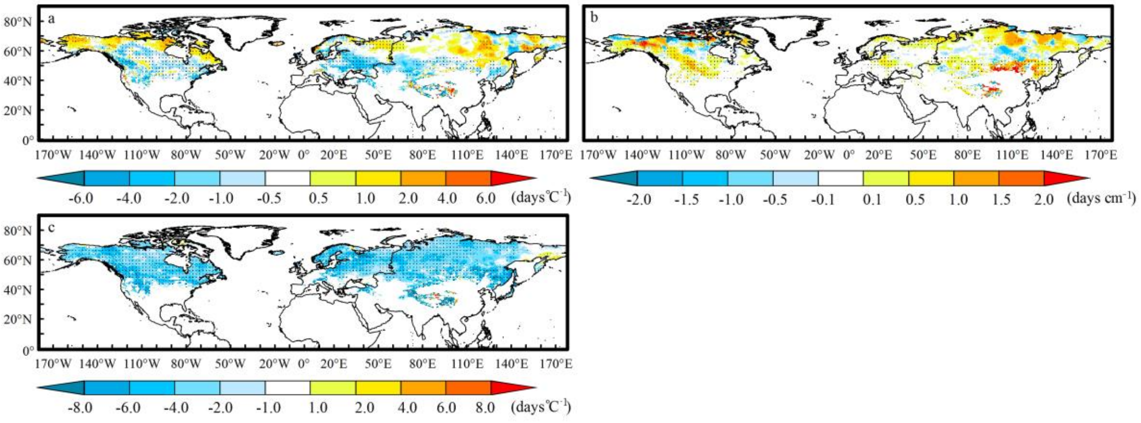

Figure S11. (**a**) Sensitivity of D_e to T_a when impacts of P_a and T_m are removed. (**b**) Sensitivity of D_e to P_a when the impacts of T_a and T_m are removed. (**c**) Sensitivity of D_e to T_m when impacts of T_a and P_a are removed. Black dots indicate correlation between D_e and T_a in **a**, D_e and P_a in **b**, D_e and T_m in **c** are significant at 90% CL. The figure was created using *ArcGIS* (version10.2.2) [S40].

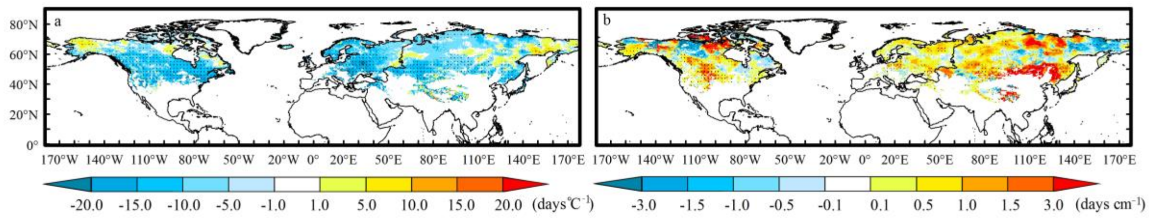

Figure S12. (**a**) Sensitivity of D_d to T_s when impacts of P_a are removed. (**b**) Sensitivity of D_d to P_a when impacts of T_s are removed. Black dots indicate correlation between D_d and T_s in **a**, D_d and P_a in **b** are significant at 90% CL. The figure was created using *ArcGIS* (version10.2.2)[S40].

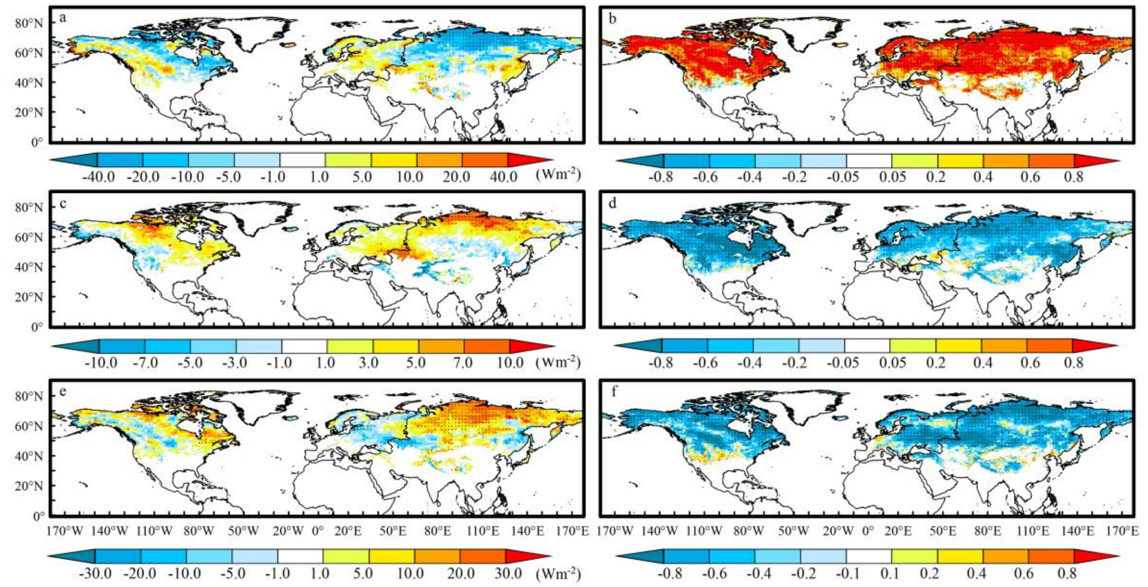

Figure S13. Clouds and Earth's Radiant Energy System (CERES) observed 13-year changes of (**a**) melting season shortwave flux, (**c**) longwave flux and (**e**) Net flux at the top of atmosphere (TOA) from 2001 to 2013. The linear correlations between snow end date (D_e) with TOA (**b**) shortwave, (**d**) longwave, and (**f**) net flux in melting seasons from 2001 to 2013. Changes are derived from the linear slope multiplied by the time span. Black dots in (**a**), (**c**), and (**e**) indicate that the changes are significant at 90% CL. Black dots in (**b**), (**d**), and (**f**) indicate that the correlation are significant at 90% CL. The figure was created using *ArcGIS* (version10.2.2)[S40].

Supplementary Tables

Table S1. Summary of snow datasets used in this study

Name	Time span	Spatial resolution	Temporal resolution	Reference/Sources
CMC	2000.09-2014.12	24 km	daily	http://nsidc.org/data/nsidc-0447
IMS	2000.09-2014.12	24 km	daily	http://nsidc.org/data/g02156
NHSCE	2000.09-2014.06	25 km	weekly	http://nsidc.org/data/nsidc-0046
MOD10C2	2000.09-2014.12	0.05°	8-day	http://nsidc.org/data/MOD10C2
NISE	2000.09-2014.12	25 km	daily	http://nsidc.org/data/nise1

Table S2. 14-year averaged values of snow onset date (D_o), snow end date (D_e), and snow duration days (D_d) in day of the year detected by five snow data sets between 32°N and 75°N from 2001 to 2014.

Data	CMC	IMS	NHSCE	MODIS	NISE	Average
D_o	291.32 (2.53)	294.72 (2.29)	301.59 (3.10)	279.49 (1.39)	289.53 (1.39)	290.53 (8.58)
D_e	130.71 (2.92)	122.79 (2.86)	122.28 (2.43)	135.67 (1.89)	104.62 (1.62)	127.86 (5.61)
D_d	196.57 (3.76)	176.88 (4.05)	174.34 (3.14)	217.91 (1.87)	164.80 (3.68)	186.10 (18.97)

The value in parentheses indicates standard deviation.

Table S3. 14-year averaged changes of snow onset date (D_o), snow end date (D_e) and snow duration (D_d) detected by five snow data sets over the NH between 32°N to 75°N from 2001 to 2014.

Data	CMC	IMS	NHSCE	MODIS	NISE
D_o	4.42 (0.06)	-0.20 (0.93)	-6.30 (0.04)	1.09 (0.43)	2.58 (0.05)
D_e	0 (0.99)	-6.26 (0)	-3.89 (0.01)	-1.47 (0.03)	-3.20 (0.37)
D_d	-0.86 (0.82)	-5.66 (0.15)	2.38 (0.45)	-2.44 (0.08)	-5.58 (0.12)

The value in parentheses indicates standard deviation in the first row and P value in the second row.

Table S4. Correlation (r) of snow onset date (D_o), snow end date (D_e) and snow duration days (D_d) z-score standardized series with the average anomaly series from the four other snow datasets.

Data	MODIS	CMC	NHSCE	IMS	NISE
D_o	0.71	0.65 (0.40)	0.67 (0.75)	0.81 (0.59)	0.73 (0.54)
D_e	0.93	0.61 (0.78)	0.65 (0.68)	0.80 (0.82)	0.71 (0.76)
D_d	0.80	0.79 (0.67)	0.79 (0.63)	0.91 (0.85)	0.68 (0.64)

The value in parentheses indicates correlations of D_o, D_e, and D_d anomalies series with the MODIS.

Table S5. The RMSE for z-score standardized snow onset date (D_o), snow end date (D_e), and snow duration days (D_d) anomalies series from each data series versus the average standardized anomaly series from the four other snow datasets.

Data	MODIS	CMC	NHSCE	IMS	NISE
D_o	0.45	0.58 (1.09)	0.51 (0.44)	0.28 (0.62)	0.50 (0.79)
D_e	0.37	0.84 (0.67)	0.79 (0.80)	0.60 (0.77)	0.72 (0.68)
D_d	0.30	0.34 (0.57)	0.43 (0.73)	0.14 (0.25)	0.56 (0.54)

The value in parentheses indicates RMSE of D_o, D_e, and D_d anomalies series with the MODIS.

Supplementary References

S1. Frei, A. et al. A review of global satellite-derived snow products. *Adv. Space Res*. **50**, 1007-1029 (2012).
S2. Déry, S. J. & Brown, R. D. Recent Northern Hemisphere snow cover extent trends and implications for the snow-albedo feedback. *Geophys. Res. Lett*. **34**, L22504 (2007).
S3. Derksen, C. & Brown, R. Spring snow cover extent reductions in the 2008-2012 period exceeding climate model projections. *Geophys. Res. Lett*. **39,** L19504 (2012).
S4. Brown, R. D. & Robinson, D. A. Northern Hemisphere spring snow cover variability and change over 1922–2010 including an assessment of uncertainty. *Clim. Chang*. **5**, 219-229 (2011).
S5. Brown, R., Derksen, C. & Wang, L. A multi-data set analysis of variability and change in Arctic spring snow cover extent, 1967–2008. *J. Geophys. Res*. **115**, D16111 (2010).
S6. IPCC. *Climate Change 2013: The Physical Science Basis. Contribution of Working Group I to the Fifth Assessment Report of the Intergovernmental Panel on Climate Change*. (Cambridge Univ. Press, 2013).
S7. Flanner, M. G., Shell, K. M., Barlage, M., Perovich, D. K. & Tschudi, M. A. Radiative forcing and albedo feedback from the Northern Hemisphere cryosphere between 1979 and 2008. *Nat. Geosci*. **4**, 151-155 (2011).
S8. P. Y. Groisman, T. R. Karl & Knight, R. W. Observed Impact of Snow Cover on the Heat Balance and the Rise of Continental Spring Temperatures. *Science*. **14**, 198-200 (1994).
S9. Fernandes, R. et al. Controls on Northern Hemisphere snow albedo feedback quantified using satellite Earth observations. *Geophys. Res. Lett*. **36**, L21702 (2009).
S10. Choi, G., Robinson, D. A. & Kang, S. Changing Northern Hemisphere Snow Seasons. *J. Clim*. **23**, 5305-5310 (2010).
S11. Whetton, R. H., Haylock, M. R. & Galloway, R. Climate change and snow-cover duration in the Australian Alps. *Clim. Chang*. **32**, 447-479 (1996).
S12. Wang, L., Derksen, C., Brown, R. & Markus, T. Recent changes in pan-Arctic melt onset from satellite passive microwave measurements. *Geophys. Res. Lett*. **40**, 522-528 (2013).
S13. Beniston, M. Variations of Snow depth and duration in the Swiss Alps over the Last 50 Years: Links to Changes in Large-scale Climate Forcing. *Clim. Chang*. **36**, 281-300 (1997).
S14. Peng, S. et al. Change in snow phenology and its potential feedback to temperature in the Northern Hemisphere over the last three decades. *Environ. Res. Lett*. **8**, 014008 (2013).
S15. Mioduszewski, J. R., A. K., R., D. A., R. & T. L., M. Attribution of snowmelt onset in Northern Canada. *J. Geophys. Res*. **119**, 9638-9653 (2014).
S16. Hall, D. K., Kelly, R. E. J., Riggs, G. A., Chang, A. T. C. & Foster, J. L. Assessment of the relative accuracy of hemispheric-scale snow-cover maps. *Ann. Glaciol*. **34**, 24-30(2002).
S17. Brasnett, B. A Global Analysis of Snow Depth for Numerical Weather Prediction. *J. Appl. Meteorol. Climatol*. **38**, 726-740 (1999).
S18. Helfrich, S. R., McNamara, D., Ramsay, B. H., Baldwin, T. & Kasheta, T. Enhancements to, and forthcoming developments in the Interactive Multisensor Snow and Ice Mapping System (IMS). *Hydrol. Process*. **21**, 1576-1586 (2007).
S19. Robinson, D. A., Dewey, K. F. & Richard R. Heim, J. Global Snow Cover Monitoring: An Update. *Bull. Amer. Meteor. Soc.* **74**, 1689-1696 (1993).
S20. Hall, D. K., Riggs, G. A. & Salomonson, V. V. Development of methods for mapping global snow cover using moderate resolution imaging spectroradiometer data. *Remote Sens. Environ*. **54**, 127-140, (1995).
S21. Nolin, A. W., Armstrong, R. & Maslanik., J. *Near-Real-Time SSM/I-SSMIS EASE-Grid Daily Global Ice Concentration and Snow Extent. Version 4.* doi:http://dx.doi.org/10.5067/VF7QO90IHZ99 (1998).
S22. Brown, R. D., Brasnett, B. & Robinson, D. Gridded North American monthly snow depth and snow water equivalent for GCM evaluation. *Atmos.-Ocean.* **41**, 1-14 (2003).
S23. Hall, D. K. & Riggs, G. A. Accuracy assessment of the MODIS snow products. *Hydrol. Process*. **21**, 1534-1547 (2007).
S24. Brown, R., Derksen, C. & Wang, L. Assessment of spring snow cover duration variability over northern Canada from satellite datasets. *Remote Sens. Environ*. **111**, 367-381 (2007).
S25. Estilow, T. W., Young, A. H. & Robinson, D. A. A long-term Northern Hemisphere snow cover extent data record for climate studies and monitoring. *Earth Syst. Sci. Data*. **7**, 137-142(2015).
S26. Menne, M. J., Durre, I., Vose, R. S., Gleason, B. E. & Houston, T. G. An Overview of the Global Historical Climatology Network-Daily Database. *J. Atmos. Oceanic Technol*. **29**, 897-910, (2012).

S27. Klein Tank, A. M. G. et al. Daily dataset of 20th-century surface air temperature and precipitation series for the European Climate Assessment. *Int. J. Climatol*. **22**, 1441-1453 (2002).
S28. Liang, S. et al. A long-term Global LAnd Surface Satellite (GLASS) data-set for environmental studies. *Int. J. Digital Earth.* **6**, 5-33 (2013).
S29. Liang, S. et al. *Global LAnd Surface Satellite (GLASS) products: Algorithms, validation and analysis*. (Springer, 2013).
S30. Moody, E. G., King, M. D., Schaaf, C. B. & Platnick, S. MODIS-Derived Spatially Complete Surface Albedo Products: Spatial and Temporal Pixel Distribution and Zonal Averages. *J. Appl. Meteorol. Climatol*. **47**, 2879-2894 (2008).
S31. He, T. et al. Greenland surface albedo changes in July 1981–2012 from satellite observations. *Environ. Res. Lett*. **8**, 044043 (2013).
S32. He, T., Liang, S. & Song, D. Analysis of global land surface albedo climatology and spatial-temporal variation during 1981–2010 from multiple satellite products. *J. Geophys. Res*. **119**, (2014).
S33. Wielicki, B. A. et al. Clouds and the Earth’s Radiant Energy System (CERES): An Earth observing system experiment. *Bull. Amer. Meteor. Soc.* **77**, 853–868 (1996).
S34. Loeb, N. G. et al. Toward Optimal Closure of the Earth's Top-of-Atmosphere Radiation Budget. *J. Clim*. **22**, 748-766 (2009).
S35. Harris, I., Jones, P. D., Osborn, T. J. & Lister, D. H. Updated high-resolution grids of monthly climatic observations - the CRU TS3.10 Dataset. *Int. J. Climatol*. **34**, 623-642 (2013).
S36. Shell, K. M., Kiehl, J. T. & Shields, C. A. Using the Radiative Kernel Technique to Calculate Climate Feedbacks in NCAR’s Community Atmospheric Model. *J. Clim*. **21**, 2269-2282, (2008).
S37. Soden, B. J. et al. Quantifying Climate Feedbacks Using Radiative Kernels. *J. Clim*. **21**, 3504-3520, (2008).
S38. Hansen, J., Ruedy, R., Sato, M. & Lo, K. Global Surface Temperature Change. *Rev. Geophys*. **48**, (2010).
S39. Barnett, T. P., Adam, J. C. & Lettenmaier, D. P. Potential impacts of a warming climate on water availability in snow-dominated regions. *Nature*. **438**, 303-309 (2005).
S40. Law, M. and Collins, *A. Getting to Know ArcGIS for Desktop, Third Edition*. (Esri Press, 2013)
